# Pneumocystis Pneumonia in Cirrhosis: An Underrecognized Fungal Infection in a Vulnerable Host

**DOI:** 10.3390/jof11070500

**Published:** 2025-07-03

**Authors:** Aaron M. Pulsipher, Michele Barnhill, Holenarasipur R. Vikram, Michael B. Gotway, Rodrigo Cartin-Ceba, Kevin Zhou, Emily R. Thompson, Andrew H. Limper, Bashar Aqel, Kealy Ham

**Affiliations:** 1 Department of Critical Care, Mayo Clinic, Phoenix, AZ 85054, USA; cartinceba.rodrigo@mayo.edu (R.C.-C.); ham.kealy@mayo.edu (K.H.); 2 Division of Gastroenterology and Hepatology, Mayo Clinic, Phoenix, AZ 85054, USA; barnhill.michele@mayo.edu (M.B.); aqel.bashar@mayo.edu (B.A.); 3 Division of Infectious Diseases and Transplant Center, Mayo Clinic, Phoenix, AZ 85054, USA; vikram.hr@mayo.edu; 4 Division of Cardiothoracic Radiology, Mayo Clinic, Phoenix, AZ 85054, USA; gotway.michael@mayo.edu; 5 Department of Internal Medicine, Mayo Clinic, Phoenix, AZ 85054, USA; zhou.kevin2@mayo.edu; 6 Division of Clinical Trials and Biostatistics, Mayo Clinic Arizona, Phoenix, AZ 85054, USA; thompson.emily3@mayo.edu; 7 Division of Pulmonary and Critical Care, Mayo Clinic, Rochester, MN 55905, USA; limper.andrew@mayo.edu

**Keywords:** cirrhosis-associated immune dysfunction, immunocompromised host, chronic liver failure, opportunistic infections, antifungal prophylaxis

## Abstract

Pneumocystis pneumonia (PCP) is a serious fungal infection affecting immunocompromised hosts. Decompensated cirrhosis leads to cirrhosis-associated immune dysfunction (CAID), a form of impaired cellular immunity that may predispose patients to opportunistic infections such as PCP. We conducted a retrospective review of 727 patients with proven or probable PCP from 2017 to 2025. Of these, 33 had decompensated cirrhosis. These patients were stratified into two groups: Cirrhosis Only (n = 16) and Cirrhosis with Additional Immunocompromising Conditions (n = 17). Among the patients with cirrhosis, the overall mortality was 48%, with the 90-day mortality reaching 57.6% (95% CI: 39.2–74.5%). Compared with those without cirrhosis, the patients with cirrhosis had a higher risk of mortality (OR: 4.08, 95% CI: 2.01–8.30, *p* < 0.001), increased intensive care unit (ICU) admission (87% vs. 42%, *p* < 0.001), and greater need for renal replacement therapy (54.6% vs. 7.5%, *p* < 0.001). These findings suggest that decompensated cirrhosis alone may represent a sufficient and underrecognized risk factor for PCP, with a high associated mortality. Given the preventable nature of this infection, future studies are needed to assess the incidence, define the risk, and investigate the role of prophylaxis in this vulnerable population.

## 1. Introduction

Pneumocystis pneumonia (PCP) is a serious opportunistic fungal infection caused by *Pneumocystis jirovecii* and has historically been associated with advanced human immunodeficiency virus (HIV), but it is now increasingly observed in non-HIV populations with impaired cellular immunity [1,2,3,4,5]. These conditions include hematologic malignancies, chemotherapy for solid organ tumors, hematopoietic stem cell transplantation, solid organ transplantation, and the use of immunosuppressive medications for autoimmune diseases [6,7,8,9,10,11,12,13]. Glucocorticoid exposure is also a well-known risk factor for PCP and is addressed in the existing prophylactic guidelines [14,15]. However, prophylactic guidelines are lacking for conditions in which the risk for PCP is less well described, such as cirrhosis.

Cirrhosis has not traditionally been recognized as a risk factor for PCP despite a growing number of case reports and observational studies in the literature [16,17]. Patients with advanced cirrhosis are susceptible to cirrhosis-associated immune dysfunction (CAID), a condition characterized by a dysregulated immune response that parallels the severity of liver disease [18,19]. Cirrhosis-associated immune dysfunction is characterized by persistent low-grade or high-grade systemic inflammation, which induces a compensatory anti-inflammatory state and subsequent immunosuppression. This immunosuppression includes lymphocyte apoptosis, functional lymphocyte defects, and T-cell anergy [19]. Additionally, depletion of CD4+ T-cells occurs due to impaired thymopoiesis and the activation of programmed cell death pathways [20]. In alcohol-related cirrhosis, natural killer cell dysfunction and attenuated responses to cytokine stimulation have also been described [21]. Collectively, these immune alterations contribute to impaired cellular immunity in decompensated cirrhosis, rendering patients susceptible to infections, including those caused by opportunistic pathogens like *Pneumocystis jirovecii*.

This study aimed to characterize the clinical features, risk factors, and outcomes of PCP in patients with decompensated cirrhosis. By highlighting this underrecognized risk, we seek to raise clinician awareness and help inform future guideline development and prophylaxis strategies.

## 2. Materials and Methods

### 2.1. Patients and Data Collection

This study received approval from the Mayo Clinic institutional review board (ID: 24-005981) and was granted a waiver of informed consent in accordance with federal regulations, as it was deemed to involve no more than minimal risk based on its retrospective design. Adult patients (≥18 years of age) across the multicenter healthcare system were initially screened for possible inclusion based on either an International Classification of Diseases (ICD) code for pneumocystis pneumonia (PCP) or a positive polymerase chain reaction (PCR) assay for *P. jirovecii* between January 2017 and February 2025.

All the cases identified through this preliminary screen underwent a detailed manual review to determine whether they met the criteria for proven or probable PCP, as defined by the European Organisation for Research and Treatment of Cancer/Mycoses Study Group Education and Research Consortium (EORTC/MSGERC) [22]. Proven PCP was defined as the microscopic identification of *P. jirovecii* on biopsy or cytologic evaluation. Probable PCP required compatible clinical symptoms or imaging findings, along with a positive PCR (bronchoalveolar lavage [BAL] or sputum) or elevated serum (1,3)-β-D-glucan (BDG). In addition, the diagnosis had to be documented by the treating team as active infection rather than colonization; patients with presumed colonization were excluded.

Our institution utilizes an FDA-cleared Fungitell Assay (Associates of Cape Cod, East Falmouth, MA, USA), a kinetic ELISA based on the modification of the Limulus Amebocyte Lysate pathway. The assay has a standard cutoff value of 80 pg/mL or more to define positivity. The PCP PCR assay used at our institution targets a region of the *cdc2* gene of *Pneumocystis jirovecii*. Unlike conventional quantitative PCR methods that rely on cycle threshold cutoffs, this assay uses a melt curve analysis to determine positivity. This assay has previously been demonstrated to have a higher specificity for active infection [23,24].

Details of concurrent infections were also collected. Coinfections were defined by the presence of an additional infection at the time of diagnosis or during treatment. Bacterial pneumonia was defined by the isolation of a pathogenic bacteria from sputum or bronchoscopic alveolar lavage (BAL) culture that was subsequently treated by the clinical team. Respiratory viral infections were diagnosed using extended PCR panels performed on nasopharyngeal swabs or BAL fluid. Bacteremia was defined by the growth of pathogenic bacteria in blood cultures. Spontaneous bacterial peritonitis was diagnosed by an ascitic fluid absolute neutrophil count of >250 cells/microL or positive bacterial cultures of ascitic fluid. Finally, Aspergillus pneumonia was defined by a positive fungal culture for a pathogenic *Aspergillus* species or the detection of *Aspergillus* antigen in serum or BAL.

After establishing that the inclusion criteria were met, the clinical data were manually abstracted from the electronic medical record and entered into a secure Research Electronic Data Capture (REDCap) database [25]. Up to 330 variables were collected for each patient, including demographic characteristics, comorbid conditions, laboratory and imaging results, and clinical outcomes. All patients with cirrhosis were independently reviewed in collaboration with a board-certified hepatologist to confirm the cirrhosis etiology and the presence of decompensation. The cirrhosis cohort was stratified into two predefined subgroups for analysis—Cirrhosis Only and Cirrhosis with Additional Immunocompromising Conditions.

### 2.2. Statistical Analysis

Continuous variables with normal distributions were summarized using mean and standard deviation (SD), while skewed variables were reported as medians with interquartile ranges (IQRs). Comparisons of continuous variables across the cirrhosis subgroups were performed using Student’s *t*-test. Categorical variables were presented as counts and percentages and compared using either the chi-square test or the Fisher’s exact test, as appropriate.

Univariate logistic regression was employed to estimate the association between cirrhosis and overall mortality compared with that for the remainder of the PCP cohort. Overall mortality was defined as all-cause death occurring during the index hospitalization for inpatients or within 30 days of the index outpatient visit. The ninety-day mortality was also assessed, and the overall survival was evaluated using Kaplan–Meier survival analysis timed from the PCP diagnosis date, with differences between groups compared using the log-rank test. A *p*-value of <0.05 was considered statistically significant for all the analyses. All the statistical analyses were conducted using SAS software, version 9.4 (SAS Institute Inc., Cary, NC, USA).

## 3. Results

A total of 727 patients met the inclusion criteria for PCP; of these, 33 had cirrhosis (Table 1). We identified 16 patients with PCP in whom decompensated cirrhosis was the only predisposing risk factor and 17 patients who had decompensated cirrhosis with additional etiologies of immunocompromise. Two patients met the criteria for proven PCP; both were receiving chronic steroid therapy. The remaining 31 patients met the criteria for probable disease. All the 33 patients had a positive PCP PCR (29 through BAL, 3 through sputum, and 1 with both). The serum (1,3)-β-D-glucan (BDG) levels were measured in 26 patients, with a median value of 324 pg/mL (IQR: 112-500). (1,3)-β-D-Glucan was positive in 22 of 26 patients (85%).

The mean age of patients with cirrhosis was 51.3 (SD 15.7) with no significant differences observed between subgroups. The majority were male (58%), and the distribution of biological sex did not vary significantly across the groups. The average Model for End-Stage Liver Disease 3.0 (MELD 3.0) score was 31.5 (SD 10.1). Patients in the Cirrhosis-Only Group had a higher average MELD 3.0 (34.9, SD: 7.9) than that of those in the Cirrhosis with Additional Immunocompromising Conditions Group (28.2, SD: 11.1) although this difference did not reach statistical significance (*p* = 0.061).

Alcohol-related liver disease was the most common etiology of cirrhosis, present in 17 patients (51.5%); of them, 11 (65%) had concurrent alcohol-associated hepatitis. Metabolic dysfunction-Associated Steatotic Liver Disease (MASLD) was the second most common cause, observed in four patients (12.1%). As shown in Table 1, the etiology of cirrhosis did not vary significantly among the subgroups. The prevalence of cirrhosis-related decompensations is also noted in Table 1. Ascites was present in 88%, hepatic encephalopathy in 72.7%, and esophageal varices in 52%, with similar rates observed across all the subgroups. Hepatorenal syndrome was more common in the Cirrhosis-Only Group (50% vs. 0%, *p* = 0.001).

The most common etiology of additional immunocompromise in this cohort was chronic steroid use, present in 12 of 16 patients (75%), including 6 patients receiving steroids for alcohol-associated hepatitis. Other etiologies of immunosuppression included hematologic malignancy undergoing chemotherapy (n = 2), human immunodeficiency virus HIV infection (n = 1), and autoimmune diseases requiring immunosuppressive therapy (n = 3).

Computed tomography (CT) imaging was available for 32 patients. All the 32 patients had ground-glass opacities, and 18 (55%) had consolidations. Pleural effusions were present in 19 (59%) and were more likely to be bilateral (n = 14, 74%) than unilateral (n = 5, 26%). Interlobular septal thickening was noted in seven (22%) patients.

Concurrent infections were common, identified in 16 of 33 patients (48.5%) (Table 2). The most frequently observed coinfection was bacterial pneumonia, affecting 24% of patients, with no significant difference between groups (*p* = 1.00). Bacteremia occurred in six patients (18.2%) and Aspergillus pneumonia in four patients (12%). Lymphopenia was present in 45% of all the cirrhotic patients, with a median absolute lymphocyte count of 1.02 × 10^9^/L (range: 0.00–4.43).

**Figure 1:**
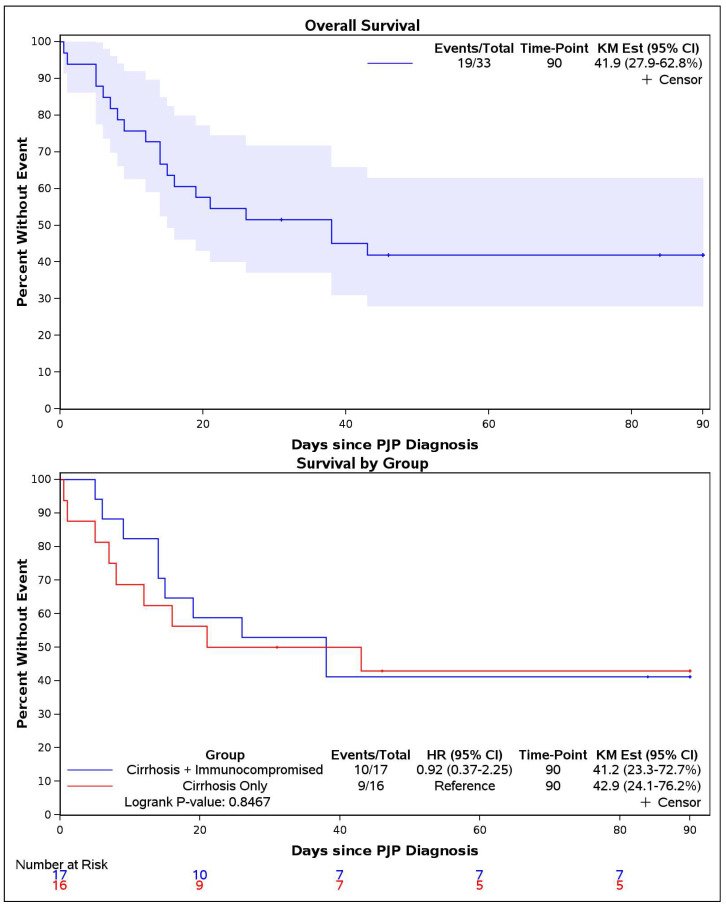
Top panel: Overall Kaplan–Meier survival curve with 95% confidence interval (blue shaded area). Bottom panel: Kaplan–Meier survival curves of the Cirrhosis-Only and Cirrhosis with Additional Immunocompromising Conditions groups. One patient died the same day as the PCR result and was given a survival of 0.5 days.

The majority of cirrhosis patients with PCP required hospital admission (30/33, 90.9%) with no significant difference in the admission rates across the subgroups (Table 3). The need for mechanical ventilation was similar between groups (46.7% vs. 35.3%, *p* = 0.51). Renal failure necessitating renal replacement therapy occurred in 18 patients (55%), with no significant difference among the subgroups. The overall mortality was 48.5% with similar rates observed between subgroups (*p*= 0.73). The ninety-day mortality had a similar trend, although this did not reach statistical significance (*p* = 1.00). Kaplan–Meier survival curves for the entire cohort and for the individual groups are shown in Figure 1. The log-rank test for differences in survival between the groups did not meet the threshold for statistical significance (*p* = 0.85).

The univariate odds ratio (OR) for overall mortality associated with cirrhosis was 4.08 (95% CI: 2.01–8.03, *p* < 0.001). This odds ratio increased to 5.69 (95% CI: 2.69–12.02, *p* < 0.001) when adjusting for age. The MELD 3.0 score was also independently associated with the overall mortality (OR 1.12;95% CI: 1.02–1.22; *p* = 0.009).

## 4. Discussion

In this multicenter retrospective cohort, we identified 33 patients with PCP who had decompensated cirrhosis; 16 of them had no additional risk factor aside from cirrhosis-associated immune dysfunction (CAID). The impaired cellular immunity characteristic of cirrhosis-associated immune dysfunction provides a biologically plausible mechanism for susceptibility to PCP, given the central role of T-cell immunity in preventing this infection. Nevertheless, patients with decompensated cirrhosis are not traditionally considered a population at risk for PCP. The presence of PCP in these patients—in the absence of other established risk factors—suggests that cirrhosis-associated immune dysfunction may represent an underrecognized state of vulnerability to opportunistic infections.

The overall mortality for patients with cirrhosis infected with PCP was 48% (16 of 33 patients), substantially higher than the 18.7% mortality observed in non-cirrhotic patients with PCP in our entire cohort. Both univariate and age-adjusted analyses substantially elevated the odds ratios for mortality, highlighting the vulnerability of cirrhotic patients. Given this high mortality, it is imperative to understand the true incidence of this infection as it is largely preventable with prophylaxis. Importantly, none of the patients with cirrhosis were receiving PCP prophylaxis, despite several being on chronic corticosteroid treatment for alcohol-associated or autoimmune hepatitis.

The delayed recognition and initiation of antimicrobial therapy likely contributed to the high mortality observed in this population. The overall empiric treatment rate for PCP across the full cohort was low (18.4%), and it was even lower among cirrhotic patients (6%). These data suggest that clinicians may not routinely consider PCP in the differential diagnosis of respiratory failure in cirrhosis. The combination of elevated mortality, low empiric treatment rates, and lack of prophylaxis supports the need for earlier risk assessment and diagnostic consideration, particularly when corticosteroids or other immunosuppressive therapies are utilized in patients with cirrhosis. Currently the guidelines for the treatment of alcoholic hepatitis do not comment on the need for PCP prophylaxis despite recommendations for prolonged courses of prednisolone [26].

To assess the current state of the literature on this topic, we performed a PubMed search using the terms “*Pneumocystis jirovecii*” OR “*Pneumocystis carinii*” OR “Pneumocystis pneumonia” AND “cirrhosis” limited to titles and abstracts. This yielded 36 results, of which 26 were unrelated, leaving only 10 articles that directly addressed pneumocystis pneumonia in patients with cirrhosis. The majority of these were case reports or small case series, underscoring the paucity of research on this association. The most robust prior study was by Peschel et al. [16], who reported a high mortality rate of 84.7% with an OR of 4.8 for mortality among the 67 patients with cirrhosis in their cohort. The higher mortality in their cohort may in part be explained by their restriction to patients in the intensive care unit (ICU), whereas our study included the entire spectrum of the illness, including those managed as outpatients. Among the patients in our cohort who required ICU care, the mortality rate for those with cirrhosis was 61.5% compared with 39.3% in the non-cirrhotic patients (*p* = 0.027). This rate rose to 76.2% (16/21) when excluding patients diagnosed during transplant evaluation.

Severity of illness was also significantly greater among cirrhotic patients. ICU admission was required in 87% (26/30) of hospitalized patients with cirrhosis compared with 42% of those without cirrhosis (*p* < 0.001). Renal replacement therapy was required in 55% (18/33) of patients with cirrhosis in contrast to only 7.5% of non-cirrhotic patients (*p* < 0.001). The rate of secondary infections was higher in the Cirrhosis-Only Group, but this did not meet statistical significance.

Six patients were diagnosed with PCP during liver transplant evaluation. These individuals generally presented with milder disease, with diagnosis prompted by abnormal chest imaging rather than by hypoxemia. Subsequent diagnostic bronchoscopy and serum (1,3)-β-D-glucan testing (positive in all four patients tested) facilitated the early recognition of the disease. Five of the six patients in this group proceeded to liver transplantation while receiving PCP therapy (mean time from antibiotic initiation to transplant: 9.8 days; range 1–18), and all survived to 90 days. None of these patients developed significant respiratory failure, and all completed their treatment courses. The sole death in this group occurred in a patient deemed unsuitable for transplant, and the death was not attributed to the PCP infection.

Taken together, these findings suggest that cirrhosis-associated immune dysfunction (CAID) may be sufficient, in the absence of other risk factors, to predispose patients to PCP. Cirrhosis-associated immune dysfunction is a well-characterized state of impaired immunity [27] and the incidence of fungal infections in this population is increasing [28]. According to the American Association for the Study of Liver Disease (AASLD), fungal infections occur in 2–16% of patients with acute on chronic liver failure [29]. Furthermore, mortality from opportunistic fungal pathogens—*Candida*, *Aspergillus*, *Pneumocystis jirovecii*, and *Cryptococcus*—is higher among cirrhotic patients than among those without cirrhosis [30].

The American Thoracic Society highlights that chronic corticosteroid use, particularly at dose ≥ 20 mg equivalent of prednisone daily for more than 2–4 weeks, significantly increases the risk of PCP [31]. The current PCP prophylaxis guidelines primarily target populations with well-defined immunosuppressive conditions such as hematologic malignancies, HIV/AIDS, and transplant recipients. However, cirrhotic patients, particularly those receiving corticosteroids, represent a high-risk group that remains largely unaddressed and likely underrecognized. Our findings, along with previously published case series [17,32,33], support the need to more precisely define the incidence of PCP in cirrhosis in order to sufficiently evaluate the inclusion of cirrhosis in future risk-based PCP prophylaxis strategies.

This study has several limitations. First, owing to its retrospective design, causality cannot be established. Second, the study population represents a complex and heterogeneously immunocompromised cohort within a single health system, potentially limiting generalizability. Third, we cannot estimate the absolute risk of acquiring PCP in patients with cirrhosis, as our analysis is restricted to those in whom the infection was diagnosed. Nonetheless, despite its modest size, this is among the largest studies to date examining PCP in patients with cirrhosis, and the findings are compelling. Improved survival in the pre-liver transplant group was likely due to liver transplantation and the completion of post-transplant PCP therapy.

Potential misclassification due to colonization is also a consideration. However, our institution uses a unique PCR assay that was designed to help differentiate between colonization and true infection [24]. This is further supported by the high rate of (1,3)-β-D-glucan positivity (85%) in this cohort. Nonetheless, the potential for false-positive (1,3)-β-D-glucan results remains a possibility, particularly in patients receiving albumin—a common intervention in cirrhosis—which is known to elevate BDG levels [34].

## 5. Conclusions

Pneumocystis pneumonia is a serious, underrecognized opportunistic infection in patients with decompensated cirrhosis. In this multicenter retrospective cohort, patients with cirrhosis-associated immune dysfunction demonstrated markedly higher rates of mortality, ICU admission, and renal failure requiring renal replacement therapy compared with non-cirrhotic patients with PCP. None of the cirrhotic patients received prophylaxis, and empiric treatment was uncommon. These findings highlight a critical gap in our understanding of host susceptibility to PCP and suggest that decompensated cirrhosis alone may confer sufficient immunosuppression to increase vulnerability to PCP—particularly in the presence of corticosteroid use.

However, given the potential risks of prophylactic therapies such as trimethoprim-sulfamethoxazole in this population, prioritization should be given to more precisely defining the true incidence of PCP in cirrhosis. Only once incidence is established can the safety, efficacy, and utility of prophylaxis be adequately evaluated. In the meantime, clinicians should maintain a heightened awareness of PCP in cirrhosis, especially in cases of new or worsening respiratory failure in this vulnerable population for whom early recognition and prompt treatment are essential.

## Figures and Tables

**Table 1:** Demographics, etiology of cirrhosis, and decompensations.

	All Groups	Cirrhosis Only	Cirrhosis + IC	*p*-Value
N	33	16	17	
Age (SD)	51.3 (15.7)	52.7 (13.0)	50 (18.3)	0.49
Male	19 (58%)	11 (68.8%)	8 (47.1%)	0.30
Caucasian	29 (88%)	15 (93.8%)	14 (82.4%)	1.00
BMI (SD)	28.2 (7.5)	28.9 (7.7)	27.5 (7.5)	0.47
MELD 3.0 (SD)	31.5 (10.1)	34.9 (7.9)	28.2 (11.1)	0.061
**Etiology of Cirrhosis**				
Alcohol	17 (51.5%)	10 (62.5%)	7 (41.2%)	0.81
MASLD	4 (12.1%)	2 (12.5%)	2 (11.8%)	
Cryptogenic	2 (9.1%)	1 (6.3)	1 (5.9%)	
Chronic Hepatitis Infection	3 (9.1%)	1 (6.3%)	2 (11.8%)	
Autoimmune Hepatitis	2 (6%)	0	2 (6.1%)	
Other ^1^	5 (12.1%)	2 (12.5%)	3 (17.7%)	
**Decompensations**				
Esophageal Varices	17 (52%)	7 (43.8%)	10 (58.8%)	0.4
Hepatic Encephalopathy	24 (72.7%)	12 (75.0%)	12 (75.0%)	1.00
Ascites	29 (88%)	15 (93.8%)	14 (82.4%)	0.60
Hepatorenal Syndrome	8 (24%)	8 (50%)	0 (0%)	0.001

^1^ Hemochromatosis, sarcoidosis, primary sclerosing cholangitis, primary biliary cirrhosis, congenital.

**Table 2:** Concurrent infections diagnosed *.

	All Cirrhosis	Cirrhosis Only	Cirrhosis + IC	*p*-Value
Any	16 (48.5%)	10 (62.5%)	6 (35.3%)	0.17
Aspergillus Pneumonia	4 (12%)	1 (6.3%)	3 (17.7%)	0.60
Bacterial Pneumonia	8 (24%)	4 (25%)	4 (23.5%)	1.00
Respiratory Viral	3 (9.1%)	2 (12.5%)	1 (5.9%)	0.56
Bacteremia	6 (18.2%)	2 (12.5%)	4 (23.5%)	0.62
Spontaneous Bacterial Peritonitis	2 (6.1%)	2 (12.5%)	0 (0%)	0.27
Other ^1^	3 (9.1%)	1 (6.3%)	2 (11.8%)	1.00

^1^ Other—coccidioidomycosis (n = 2), histoplasmosis and blastomycosis (n = 1). * Coinfections were identified as clinically significant infections identified at or during the treatment course for PCP.

**Table 3:** Clinical data and outcomes.

	All Cirrhosis	Cirrhosis Only	Cirrhosis + Immunocompromise	*p*-Value
N	33	16	17	
BDG (SD)	293.8 (200.7)	288.3 (191)	299.3 (168)	0.89
INR (SD)	2.59 (1.8)	2.49 (0.9)	2.59 (2.4)	0.88
Bilirubin (SD)	14.7 (12.8)	16.7 (13.4)	12.7 (12.3)	0.39
Albumin (SD)	3.1 (0.6)	3.24 (0.53)	2.95 (0.58)	0.14
Absolute Lymph Count (IQR)	1.02 (0.57–1.22)	0.93 (0.64–1.20)	1.16 (0.44–1.62)	0.54
Admission	30 (90.9%)	16 (100%)	14 (82.4%)	0.23
Hospital LOS (IQR)	19.5 (15)	21.5 (13.5–30.5)	19.0 (11–26)	0.20
ICU	26 (86.7%)	15 (93.8%)	11 (78.6)	0.32
ICU LOS (IQR)	11 (12)	9 (5–20)	12 (6–18)	0.78
Oxygen at Diagnosis				
*Room Air*	8 (25%)	4 (26.7%)	4 (23.5%)	0.51
*NC*	5 (15.6%)	3 (20%)	2 (11.8%)	
*HHFNC/BiPAP*	6 (18.8%)	1 (6.7%)	5 (29.4%)	
*Mechanical Ventilation*	13 (40.6%)	7 (46.7%)	6 (35.3%)	
Renal Failure with Renal Replacement	18 (55%)	10 (62.5%)	8 (47.1%)	0.49
Overall Mortality	16 (48.5%)	7 (43.8%)	9 (52.9%)	0.73
90-Day Mortality	19 (57.6%)	9 (56.3%)	10 (58.8%)	1.00

Abbreviations: BDG—beta-D-glucan, INR—international normalized ratio, LOS—length of stay, ICU—intensive care unit, NC—nasal cannula, HHFNC—heated high-flow nasal cannula, BiPAP—bilevel positive airway pressure.

## Data Availability

The data supporting the findings in this study are not publicly available due to patient confidentiality and institutional restrictions.

## Abbreviations

The following abbreviations are used in this manuscript:

PCP	Pneumocystis pneumonia
CAID	Cirrhosis-associated immune dysfunction
HIV	Human immunodeficiency virus
BAL	Bronchoalveolar lavage
BDG	(1,3)-β-D-Glucan
MASLD	Metabolic dysfunction-associated steatotic liver disease
ICU	Intensive care unit
